# Population Structure of Clinical *Pseudomonas aeruginosa* from West and Central African Countries

**DOI:** 10.1371/journal.pone.0107008

**Published:** 2014-09-04

**Authors:** Pascal Cholley, Roughyatou Ka, Christophe Guyeux, Michelle Thouverez, Nathalie Guessennd, Beniam Ghebremedhin, Thierry Frank, Xavier Bertrand, Didier Hocquet

**Affiliations:** 1 Laboratoire d'Hygiène Hospitalière, UMR 6249 CNRS Chrono-environnement, Université de Franche-Comté, Centre Hospitalier Universitaire, Besançon, France; 2 Laboratoire de Bactériologie, Centre Hospitalier National Universitaire de Fann, Dakar, Senegal; 3 Département d'Informatique des Systèmes Complexes, UMR 6174 CNRS, Université de Franche-Comté, Belfort, France; 4 Institut Pasteur, Abidjan, Ivory Coast; 5 Otto-von-Guericke University, Magdeburg, Germany; 6 Institut Pasteur, Bangui, Central African Republic; 7 Centre de Ressources Biologiques Ferdinand Cabanne – Filière microbiologie, Centre Hospitalier Régional Universitaire, Besançon, France; Institut National de la Recherche Agronomique, France

## Abstract

**Background:**

*Pseudomonas aeruginosa* (*PA*) has a non-clonal, epidemic population with a few widely distributed and frequently encountered sequence types (STs) called ‘high-risk clusters’. Clinical *P. aeruginosa* (clin*PA*) has been studied in all inhabited continents excepted in Africa, where a very few isolates have been analyzed. Here, we characterized a collection of clin*PA* isolates from four countries of West and Central Africa.

**Methodology:**

184 non-redundant isolates of clin*PA* from hospitals of Senegal, Ivory Coast, Nigeria, and Central African Republic were genotyped by MLST. We assessed their resistance level to antibiotics by agar diffusion and identified the extended-spectrum β-lactamases (ESBLs) and metallo-β-lactamases (MBLs) by sequencing. The population structure of the species was determined by a nucleotide-based analysis of the entire *PA* MLST database and further localized on the phylogenetic tree (*i*) the sequence types (STs) of the present collection, (*ii*) the STs by continents, (iii) ESBL- and MBL-producing STs from the MLST database.

**Principal Findings:**

We found 80 distinct STs, of which 24 had no relationship with any known STs. ‘High-risk’ international clonal complexes (CC155, CC244, CC235) were frequently found in West and Central Africa. The five VIM-2-producing isolates belonged to CC233 and CC244. GES-1 and GES-9 enzymes were produced by one CC235 and one ST1469 isolate, respectively. We showed the spread of ‘high-risk’ international clonal complexes, often described as multidrug-resistant on other continents, with a fully susceptible phenotype. The MBL- and ESBL-producing STs were scattered throughout the phylogenetic tree and our data suggest a poor association between a continent and a specific phylogroup.

**Conclusions:**

ESBL- and MBL-encoding genes are borne by both successful international clonal complexes and distinct local STs in clin*PA* of West and Central Africa. Furthermore, our data suggest that the spread of a ST could be either due to its antibiotic resistance or to features independent from the resistance to antibiotics.

## Introduction

*Pseudomonas aeruginosa* is an important opportunistic human pathogen causing infection in patients with impaired immune systems [1]. Its intrinsic resistance to many classes of antibiotics and its capacity to acquire resistance to almost all effective antibiotics during treatment render infections with this microorganism very difficult to treat [2], [3]. Resistance to β-lactams is of particular concern in clinical practice. Hence, high-level resistance to these compounds is achieved by AmpC cephalosporinase overproduction or by the production of acquired β-lactamases with an extended spectrum (i.e., extended-spectrum-β-lactamases [ESBLs], metallo-β-lactamases [MBLs], and extended-spectrum oxacillinases) [4].

From a limited number of studies often focused on antibiotic-resistant isolates, it appears that *P. aeruginosa* has a non-clonal, epidemic population with a few widely distributed and frequently encountered sequence types (STs) called ‘high-risk clusters’ [5]–[10]. The population of clinical *P. aeruginosa* has been studied in Europe, Asia, Oceania, North and South Americas. Although West and Central Africa comprises large city agglomerations, little is known about the resistance level to antibiotics and the epidemiology of clinical *P. aeruginosa*. As proof, only 0.5% (8 isolates out of 1542) of the *P. aeruginosa* multilocus sequence typing (MLST) database concerns African isolates [11].

Large-scale epidemiological studies rely on the analysis of MLST developed by Curran et al. in 2004. This method is a reproducible manner to compare isolates that are temporally and geographically distant and handled by different laboratories, allowing a better knowledge of the global epidemiology in *P. aeruginosa* [7]. Quantifying the diversity of specific house-keeping genes by MLST analyses is a powerful approach for understanding the evolution of the core genome and the processes that shape the species biodiversity.

The aim of this study was to characterize a collection of clinical *P. aeruginosa* isolates from four countries of West and Central Africa. We assessed the resistance level to antipseudomonal agents and identified the β-lactamases with an extended-spectrum in the resistant isolates. We also identified all their STs and compared their distribution in a global phylogenetic tree built with all the currently defined STs described worldwide.

## Material and Methods

### Isolate collection

**Figure 1:**
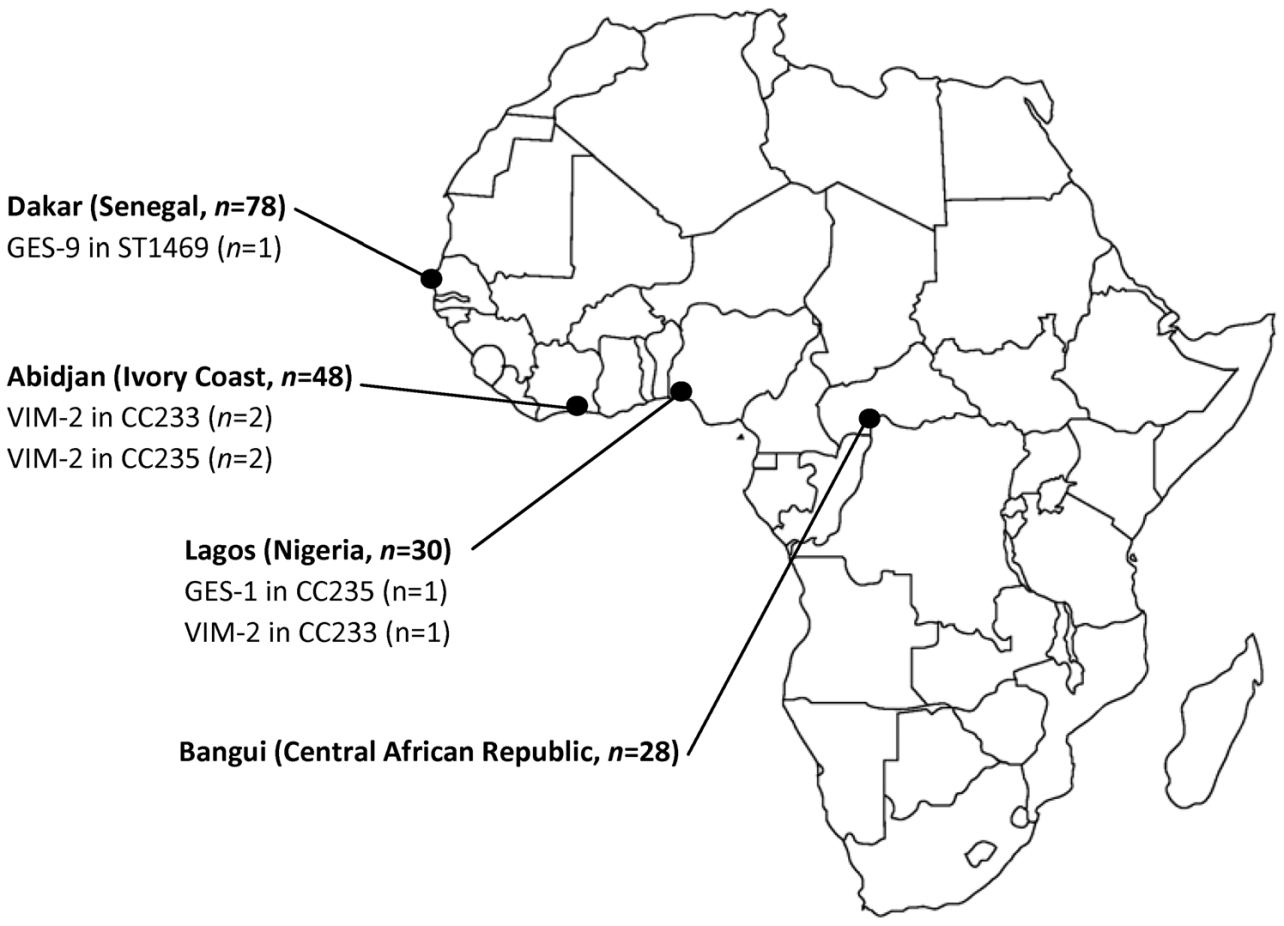
Map of Africa showing the four cities included in the study, the number of *Pseudomonas aeruginosa* clinical isolates, and the localization of ESBL- and MBL-producing isolates.

*P. aeruginosa* non-redundant clinical isolates were collected between 2002 and 2012 from patients hospitalized in the University Hospital of Fann (Dakar, Senegal, *n* = 78), in the Centre National de Référence des Antibiotiques de Côte d'Ivoire (Institut Pasteur, Abidjan, Ivory Coast, *n* = 48), in the Department of Medical Microbiology and Parasitology of the University of Lagos (Lagos, Nigeria, *n* = 30), and in the Institut Pasteur of Bangui (Bangui, Central African Republic, *n* = 28) for a total of 184 isolates. All the isolates were stored in Brain-Heart Infusion – Glycerol broth at −80°C and managed at the Centre de Ressources Biologiques Ferdinand Cabanne at the University Hospital of Besançon. Data were entirely anonymised throughout the entire study and thus ethical approval was not needed. Figure 1 represents the geographical origin of the isolates. All isolates were identified by matrix-assisted laser desorption ionization-time of flight mass spectrometry (MALDI-TOF MS) with a Microflex LT (Bruker Daltonik GmbH, Bremen, Germany), according to the manufacturer procedures [12].

### Antimicrobial susceptibility testing and β-lactamase identification

We assessed the activity of 9 antibiotics from four different classes (non-carbapenem β-lactams: cefepime, piperacillin-tazobactam, ticarcillin, ceftazidime, aztreonam; carbapenems: imipenem; aminoglycosides: tobramycin, amikacin; fluoroquinolones: ciprofloxacin) against the *P. aeruginosa* isolates by the disk diffusion method recommended by European Committee on Antimicrobial Susceptibility Testing (EUCAST, 2013 [13]). Here, all non-susceptible isolates were considered as resistant. We also identified extended-spectrum β-lactamases (ESBLs) and metallo-β-lactamases (MBLs) in isolates resistant to ceftazidime by the phenotypic method described elsewhere [14]. For isolates considered positive by this approach, the involved enzymes were identified by PCR and sequencing with primers targeting ESBL- and MBL-encoding genes [15].

### Genotyping by pulsed-field gel electrophoresis (PFGE)

The clonality of all the isolates was investigated by PFGE, with *Dra*I digestion, as previously described [16]. GelCompar software was used for cluster analysis (Applied Maths, Kortrijk, Belgium). The Dice correlation coefficients were grouped and the UPGMA clustering algorithm was used to depict the groups as a dendrogram. According to international recommendations, we clustered isolates that give PFGE patterns with ≤3 fragment differences in the same pulsotype (PT) [17]. We further genotyped by multi-locus sequence typing one isolate per PT.

### Genotyping by multi-locus sequence typing (MLST)

MLST was performed according to the protocol of Curran et al. modified by van Mansfeld et al. [18]. Nucleotide sequences were determined for internal fragments of the *acsA*, *aroE*, *guaA*, *mutL*, *nuoD*, *ppsA,* and *trpE* genes and were compared with sequences in the *P. aeruginosa* MLST website (http://pubmlst.org/paeruginosa) for the assignment of allele numbers and sequence types (ST) [11]. Clonal complexes (CCs) were defined with the software START2 as a group of STs sharing at least 5 loci [19].

### Analysis of MLST data

In order to build a dendrogram with the 1595 STs available at the time of the study (including the new ST described in this collection), we concatenated the sequences of 7 MLST genes to form a 2,882-bp sequences alignment, defining 664 polymorphic positions. The best-fit nucleotide substitution model for this data was GTR+G+I, as determined with jModelTest 0.1.1 [20]. We used the *Pseudomonas fluorescens* Pf0-1 as the outgroup [21]. Maximum likelihood tree was constructed with RAxML 7.2.8 [22] and visualized with Dendroscope 3.2.10 [23]. In every case, 1000 bootstrap repetitions gave values above 900 for most branches.

## Results

### Genotyping of the collection

We genotyped all the 184 clinical isolates from the four West and Central Africa countries by PFGE. We obtained 107 different PTs. As PFGE typing is more discriminatory than MLST, we assumed here that all the isolates that give PTs with ≤3 fragment differences shared the same ST (Cholley *et al.*, submitted manuscript). We therefore determined the MLST profiles of one isolate for each of the 107 PTs. The ST of all the isolates is given in the Table S1. We found 80 distinct STs. Among them, 47 were already in the MLST database while the other 33 (from ST1437 to ST1493) were new [11]. Nine of these new STs belonged to widespread clonal complexes: ST1461 and ST1477 belonged to the CC195, the ST1467, ST1479, ST1481, ST1482 and ST1484 belonged to the CC244, the ST1466 belonged to the CC274 and ST1472 belonged to the CC233. Besides, 24 new STs were singletons, meaning that they share less than 5 loci with known STs. In our collection, we sought to identify isolates of successful international STs or CCs (i.e. that are found worldwide). We identified from the MLST database 15 intercontinental STs or CCs (isolated in patients from ≥3 continents among Europe, North and South Americas, Asia and Oceania): ST17, ST27, CC155, ST179, CC195, CC233, CC235, CC244, ST254, CC274, ST277, CC395, ST446, ST560, and CC654 [11]. These STs and CCs were not specific to the human host and have also been described in the environment. Nine of these widespread clinical STs or CCs were also present in West and Central Africa. Hence, CC155, CC244, CC235, CC195, CC274, CC233, CC395, CC654, and ST277 were represented by 23, 19, 8, 4, 4, 3, 3, 3, and 1 isolate, respectively in the present collection.

### Resistance to antibiotics and production of extended-spectrum β-lactamases (ESBLs) and metallo-β-lactamases (MBLs)

Table 1 details the resistance rates to antibiotics of the isolates broken down by the country of origin. The antibiotic resistance profile of all the isolates is detailed in the Table S1. The resistance rates to the tested antibiotics remained low (≤10%) for isolates from Senegal and Central African Republic, with the exception of the higher resistance rate to ticarcillin in the latter country (25%). This contrasts with the much higher resistance rate of isolates from Ivory Coast and Nigeria. In these countries, 23–40% of the isolates were resistant to penicillins (ticarcillin and piperacillin/tazobactam), 13–30% were resistant to cephalosporins (cefepime and ceftazidime), 10–20% were resistant to aminoglycosides (tobramycin and amikacin), and 27% were resistant to ciprofloxacin.

**Table 1:** Characteristics of clinical isolates from West and Central Africa (n = 184), by country.

			Resistance rate (%) or resistance status^a^								
Country of isolation	Number of isolates	ST or CC	Tic	Tzp	Caz	Fep	Atm	Imp	Amk	Tob	Cip
Senegal											
Total	78	-	7.7	1.3	1.3	1.3	0^b^	0	1.3	5.1	3.8
GES-9-producing isolate	1	1469	R	S	R	R	I	S	R	R	R
Central African Republic											
Total	28	-	25.0	10.7	7.1	0	0^b^	0	0	7.1	7.1
Ivory Coast											
Total	48	-	29.2	22.9	14.6	12.5	2.1^b^	16.7	16.7	22.9	27.1
VIM-2-producing isolates	4	244 (n = 2), 233 (n = 2)	100	100	100	100	100^b^	100	100	100	100
Nigeria											
Total	30	-	40.0	26.7	20.0	30.0	3.3^b^	10	20	20	26.7
GES-1-producing isolate	1	235	R	R	R	R	I	R	R	R	R
VIM-2-producing isolate	1	233	R	R	R	R	I	R	R	R	R

a Resistance rate or resistance status were defined according to the 2013 recommendations of the European Committee on Antimicrobial Susceptibility Testing (EUCAST, 2013 [13]). Tic: ticarcillin; Tzp: Pipercillin-tazobactam; Caz: Ceftazidime; Fep: Cefepime; Atm: Aztreonam; Imp: Imipenem; Amk: Amikacin; Tob: Tobramycin; Cip: Ciprofloxacin. Here, all non-susceptible isolates were considered as resistant.

b Only isolates with an inhibition diameter around the disk of aztreonam (30 µg) <16 mm were considered as resistant.

Eleven out of the 184 isolates collected were resistant to imipenem (Ivory Coast, *n* = 8; Nigeria, *n* = 3). We identified the metallo-β-lactamase VIM-2 in 5 of them. The remaining isolates did not produce any carbapenemase and were most probably resistant to imipenem after mutations in the gene encoding OprD or the down-regulation of the production of this porin [24]. Three VIM-2-producing isolates belonged to the CC233 (Ivory Coast, *n* = 2; Nigeria, *n* = 1) and two belonged to the CC244 (Ivory Coast, *n* = 2).

Sixteen isolates of the whole collection were resistant to ceftazidime. They were isolated mostly in Ivory Coast (*n* = 7) and Nigeria (*n* = 6). One CC235 isolate from Nigeria produced GES-1 and one ST1469 isolate from Senegal produced GES-9. Of note, none of the 28 isolates collected in Central African Republic produced β-lactamase with an extended-spectrum.

Interestingly, most of the isolates (17 out of 25) that displayed a multi-drug resistant phenotype according to the consensus definition of Magiorakos *et al.* (i.e., resistance to ≥1 agent in ≥3 antimicrobial categories) belonged to the intercontinental clonal complexes CC233 (*n* = 3), CC235 (*n* = 5), CC244 (*n* = 3), CC393 (*n* = 3), and CC654 (*n* = 3) [25]. These multi-drug resistant isolates mostly came from Nigeria (*n* = 11) and Ivory Coast (*n* = 10).

### Spread of wild-type and antibiotic-resistant STs and CCs in West and Central Africa

Among the collection, seven STs were represented by more than 5 isolates. CC155 spread in Senegal with 23 isolates with a wild-type susceptibility phenotype. We retrieved the CC244 in the four countries studied. Of the 19 CC244 isolates, 4 were multi-drug resistant (among which 2 isolates produced VIM-2) while the remaining 15 displayed a wild-type susceptibility phenotype. The CC235 was represented by 8 isolates found in all countries except Senegal. Most of the CC235 isolates (6 out of 8) were resistant to ≥1 β-lactam, to ≥1 aminoglycoside, and to ciprofloxacin, and one Nigerian isolate produced the class A ESBL GES-1. All the ST699 isolates found in the collection of Nigeria, Senegal, and Ivory Coast (*n* = 7) were susceptible to all the antibiotics tested. The seven ST856 isolates were retrieved in Nigeria. We also found 7 isolates of CC393 coming from Nigeria (*n* = 6) or Central African Republic (*n* = 1). Finally, the 5 ST1464 isolates found in Senegal were fully susceptible to all the tested antibiotics.

### Population structure of clinical Pseudomonas aeruginosa in West and Central Africa

**Figure 2:**
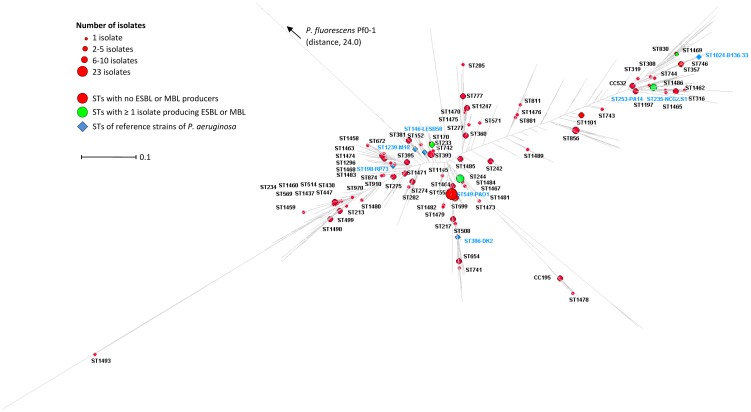
Distribution of the STs and CCs of the 184 clinical isolates of *Pseudomonas aeruginosa* isolated in West and Central Africa on a dendrogram built with the data all known STs (*n* = 1595). STs without any isolates producing ESBL or MBL were represented with red spots. STs represented by ≥1 isolate producing ESBL or MBL were represented with green spots. STs of reference isolates are represented with blue diamonds. The dendrogram is based on the alignment of the concatenated sequences of the *acsA*, *aroE*, *guaA*, *mutL*, *nuoD*, *ppsA* and *trpE* genes (forming an artificial 2,882-bp sequence) of the 1595 STs of the *P. aeruginosa* MLST database in July 2013 [11]. See Material and methods section for details.

**Figure 3:**
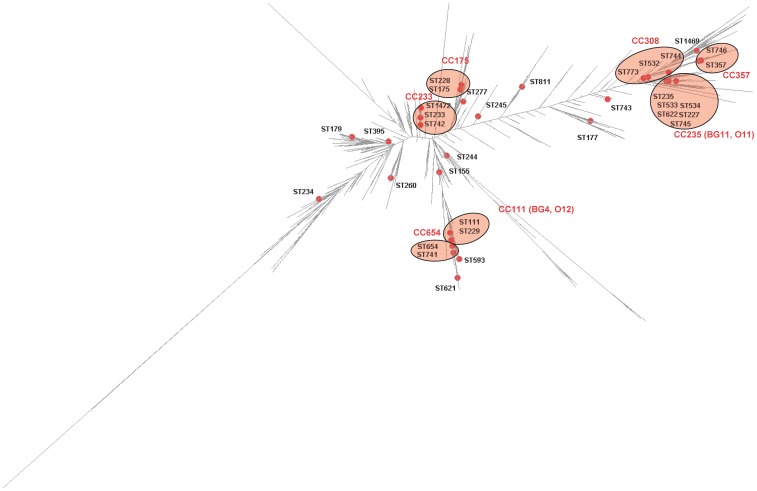
Distribution of the STs and CCs of the *Pseudomonas aeruginosa* containing isolates producing either extended-spectrum β-lactamase or metallo-β-lactamase defined in the following references [5], [28], [45], [46] on a dendrogram built with the data all known STs (*n* = 1595). ESBL- and MBL-producers were represented with red spots. See Figure 2 legend for the tree construction details. Pink zones surround STs that belong to the same clonal complex.

We then spotted the West and Central African *P. aeruginosa* isolates on the maximum likelihood (ML) tree built from the entire *P. aeruginosa* PubMLST isolate database (1595 STs described on July 2013) [11]. The ML tree displayed a bush-like structure (Figure 2). Clonal complex is defined as a group of STs in a population that share ≥5 alleles. In the vast majority of the cases, the STs grouped in one CC clustered on the ML tree (Figure 3). For instance, the STs that belong to the CC235 (e.g., ST235, ST533, ST534, ST622, ST227, and ST745) clustered on the ML tree. In two cases, STs from the same CC were scattered throughout the ML tree. Hence, ST743 and ST811 were distant from their respective predicted founders ST233 and ST155 (Figure 3).

**Figure 4:**
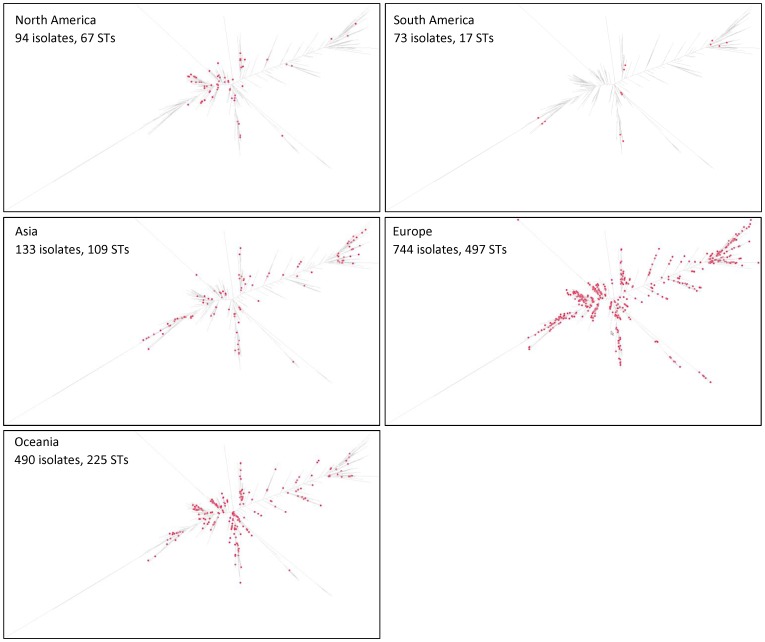
Distribution of the STs of clinical isolates of *Pseudomonas aeruginosa* on a dendrogram built with the data all known STs (*n* = 1595), broken down by their continent of origin. The geographical origin of each ST was extracted from the *P. aeruginosa* MLST database in July 2013 [11].

The 80 STs and CCs found in the four African countries (red dots on the Figure 2) were scattered throughout the tree with no particular association with a specific phylogroup. The STs or CCs that included ESBL- or MBL-producers (CC233, CC235, CC244, and ST1469) also were scattered throughout the ML tree (green dots on the Figure 2). We also repeated this experiment with the clinical STs and CCs isolated from the 5 other inhabited continents (Figure 4). Here again, the STs and CCs from a given continent were scattered throughout the global ML tree.

## Discussion

Here we analyzed the genetic diversity and the population structure of 184 clinical *P. aeruginosa* isolates found in West and Central Africa and identified the STs that produced β-lactamases with an extended-spectrum (ESBL and MBL). We found that β-lactamases with an extended-spectrum in this region are mostly borne by successful international clonal complexes but also by distinct local STs. Our data also suggest a very limited association between a genotype and a region.

### Epidemiology of ESBL- and MBL-producers

VIM-2 was the only carbapenemase found in our collection, confirming that this enzyme is the dominant metallo-β-lactamase over the world and that it is well established in the six populated continents [26]. Here, the *bla*_VIM-2_ was borne by the international complexes CC233 (*n* = 3) and CC244 (*n* = 2). The presence of VIM-2 has been established in Africa. Hence, a ST1488 isolate harboring *bla*_VIM-2_ has been reported in Abidjan (Ivory Coast) [27]. ST1488 belongs to the CC308, already associated with *bla*_MBL_ [5]. Besides, a CC233 isolate producing VIM-2 has already been described in Norway and associated with repatriation from Ghana [28]. In our collection, the three CC233 isolates (obtained from Nigeria and Ivory Coast) produced VIM-2. This confirms the presence of the VIM-2 enzyme in western African countries of the Gulf of Guinea. The intercontinental CC244 that has been described in Europe, Russia, China, Australia, and Brazil has only been associated with *bla*_PER-1_ in Poland [11], [29]. It is worth noting that we found CC244 isolates in the four African countries and that the majority of these isolates (15 out of 19) were fully susceptible to all the antibiotics tested. We also reported the production of an Ambler class A β-lactamase GES-1 in a CC235 isolate in Senegal. CC235 has been identified worldwide, in association with the carbapenemases VIM, IMP, FIM, and NDM [30]. This clonal complex falls into the CC/BURST Group 11 (BG11) with a serotype O11. GES-1-producing CC235 isolates have also been reported in Spain (Figure 3) [31]. Interestingly, all the CC235 isolates reported here (*n* = 8) were resistant to at least one antibiotic family. Finally, the last ESBL producer was the unrelated ST1469 found in Senegal that carried *bla*_GES-9_ (Figure 2). The gene *bla*_GES-9_ has only been reported once in an isolate of *P. aeruginosa* from a patient hospitalized in France [32]. These data indicated that the presence of β-lactamases with an extended-spectrum in West and Central Africa is due to both the presence of successful international clonal complexes (CC233, CC235, and CC244) and that of distinct unrelated STs (ST1469).

### Resistance patterns of intercontinental clonal complexes

Many epidemiological studies focused on multidrug-resistant isolates. Consequently, the vast majority of the isolates genotyped with MLST method are representatives of multi-drug resistant and outbreak STs. All microbiologists are encouraged to submit not only new allele sequences but also isolate information from already-known allele to the MLST databases so as it becomes a comprehensive resource for global epidemiology of this species. However, the submission of data for isolates belonging to already-known STs is not the rule. This constitutes one limitation of the databases. The spread of the multidrug-resistant intercontinental STs could be due either to their resistance pattern that favours them when the antibiotic pressure is high or to their intrinsic traits, independently of the antibiotic resistance. A high-level resistance to major anti-pseudomonal agents can be the result of the acquisition of foreign resistance genes such as those encoding carbapenemase or of mutations that trigger chromosomal resistance mechanisms. To the best of our knowledge, the data published so far could hardly answer this question. One strong point of our study is the absence of selection biases of the clinical isolates. Hence, we genotyped every isolate of the collection whatever its resistance phenotype. All the CC233 and CC235 isolates (*n* = 3 and 8, respectively) that we tested were multidrug-resistant, with the exception of two isolates resistant only to carboxypenicillins. Cabot et al. confirmed the absence of these clonal complexes in a collection of multi-susceptible clinical isolates in Spain [33]. In contrast, all the isolates belonging to CC155 (*n* = 23), CC195 (*n* = 4), CC395 (*n* = 3) were fully susceptible to all the antipseudomonal compounds tested. However, CC155 associated with *bla*_VIM-2_ has been reported in Spain, and multi-drug resistant CC395 was countrywide-distributed in Hungary or epidemic in a French University Hospital [34], [35].

CC244 isolates that carry *bla*_PER-1_ or *bla*_VEB-1_ have been reported in Poland and in Bulgaria [29], [36]. We also found, in our collection, isolates producing the VIM-2 carbapenemase (*n* = 2) even though most of the isolates of this clonal complex (15 out of 19) displayed a fully susceptible profile. Similarly, although the CC274 was also identified within multidrug-resistant isolates circulating in Spain [37], we found that the majority of the representatives of this clonal complex in our collection were of wild-type susceptibility pattern.

Beside intercontinental clonal complexes, we observed the spread of fully susceptible STs in West and Central African countries. Hence, seven ST699 isolates were found in Ivory Coast, Nigeria, or Senegal and four ST1464 isolates were found in Senegal. Altogether, these data could suggest that the spread of a ST among patients can be the result either (*i*) to its antibiotic resistance (e.g., CC235 and CC233) or (*ii*) to features independent from the resistance to antibiotics (e.g., CC155, CC244, CC195, CC274, and CC395). These latter features could favour human infection or colonisation directly with a better adaptation to the host (e.g. through a better adhesion to eukaryotic cells, higher biofilm formation and virulence), or indirectly with a higher frequency in environmental settings. Further studies are needed to clarify this point.

### Population structure of and acquisition of extended-spectrum β-lactamases by P. aeruginosa

We sought to determine whether the STs of clinical isolates of *P. aeruginosa* found in the West and Central Africa clustered on the global dendrogram of the 1595 currently defined STs (Figure 2). The analysis of MLST data relies on two strategies: allele- or nucleotide-based methods. The former is fast, simple, and very popular, but cannot distinguish between single-based changes in multiple loci versus multiple mutations in the same loci. It therefore disregards much of the evolutionary information contained at the nucleotide level. Nucleotide-based methods are more accurate and robust but require bioinformatics computer skills [38]. Figure 2 represents the ML tree produced by the alignment of the concatenated gene sequences of the currently known STs. Its bush-like structure confirmed that *P. aeruginosa* has a non-clonal structure [6]–[10]. The Figure 2 clearly shows the absence of any population substructures, confirming that *P. aeruginosa* evolves as a single cohesive genetic group. This, together with the large distance between the tree root and the representative of the close species *P. fluorescens* Pf0-1 taken as outgroup confirmed that *P. aeruginosa* conformed to a clear, compact, and well defined species [39].

As the 80 STs identified our isolates were scattered throughout the ML tree, they were highly representative of the global *P. aeruginosa* population. This suggests no evidence of STs or clades specific to a region. We also spotted on the global ML tree the STs found on the five other inhabited continents (Figure 4) [11]. Although the number of STs submitted by continent is uneven (e.g., 497 in Europe and 17 in South America), it also suggests the poor association between a phylogroup and a continent. Using other methods (eBURST and calculation of genetic diversity index), Kidd et al. also showed no significant difference between the genetic diversity of STs found in Queensland (Australia) and the global *P. aeruginosa* ST dataset [10], [11].

Standard phylogenetic methods assume a lack of recombination defined as the exchange of genetic information between two nucleotide sequences. Although the recombination plays a major role in the evolutionary history of *P. aeruginosa* genome, the housekeeping genes used for MLST are presumably not subjected to these mutational events [40]. However, the phylogenetic tree based on these data should be analyzed with caution. In addition, MLST loci such as *acs*, *guaA* and *mutL* underlie a high mutation rate, particularly in isolates associated with chronic infection such as cystic fibrosis [41]–[43]. Consequently, it is likely that the genetic diversity of the species is lower than that calculated from the MLST data.

### Genetic diversity of P. aeruginosa producing β-lactamases with an extended-spectrum

Although *bla*_MBL_ or *bla*_ESBL_ genes can be borne by many STs over the world, some successful international clonal complexes are frequently multidrug-resistant and their representatives are more prone to produce ESBLs and MBLs [5]. Members of these epidemic lineages have a serotype O11 and O12. The O12 and O11 lineages fall into the *P. aeruginosa* CC/BURST Group 4 (BG4) and BG11 and embrace STs that cluster tightly in the ML tree (Figure 3). We confirm here that MBL and ESBL-encoding genes are mostly borne by successful international CC233, CC235, and CC244 as previously observed in other countries [28], [30], [44]. The graphical representation on the global ML tree of all the STs defined so far as potential MBL- or ESBL-producers suggested that the acquisition of this resistance enzyme has a very limited association with a genotype [5], [28], [45], [46](Figure 3).

In conclusion, we found that the resistance rates of clinical isolates to antibiotics remained low in Senegal and Central African Republic. This contrasts with the much higher resistance rate of clinical isolates from Ivory Coast and Nigeria. Our data showed that, in *P. aeruginosa*, the β-lactamases with an extended-spectrum in West and Central Africa are mostly borne by successful international clonal complexes but also by local distinct STs. We suggest that the spread of a ST can be the result either to its antibiotic resistance or to features independent from the resistance to antibiotics.

## Supporting Information

Table S1 **Country of origin, sequence type and antibiotic resistance profile of the 184 clinical isolates of** ***P. aeruginosa.***
(XLS)
